# Thiazolidinedione Use and Risk of Bacterial Enteric Infection Among Adults With Type 2 Diabetes Mellitus

**DOI:** 10.1093/ofid/ofag457

**Published:** 2026-07-23

**Authors:** Morgan Birabaharan, David C Kaelber, Victor Nizet, Amir Zarrinpar

**Affiliations:** Division of Infectious Diseases and Global Public Health, University of California, San Diego, La Jolla, California, USA; Departments of Pediatrics, Internal Medicine, Population and Quantitative Health Sciences, Case Western Reserve University and the Center for Clinical Informatics Research and Education, MetroHealth System, Cleveland, Ohio, USA; Division of Host-Microbe Systems and Therapeutics, Department of Pediatrics, UC San Diego School of Medicine, La Jolla, California, USA; Skaggs School of Pharmacy and Pharmaceutical Sciences, University of California, San Diego, La Jolla, California, USA; Division of Gastroenterology, University of California, San Diego, La Jolla, California, USA; Center for Microbiome Innovation, University of California, San Diego, La Jolla, California, USA; Division of Gastroenterology, Jennifer Moreno Department of Veterans Affairs Medical Center, La Jolla, California, USA; Institute of Diabetes and Metabolic Health, University of California, San Diego, La Jolla, California, USA; Shu Chien-Gene Lay Department of Bioengineering, University of California San Diego, La Jolla, California, USA

**Keywords:** bacterial enteric infection, colonization resistance; electronic health records, pharmacoepidemiology, PPAR-γ, Thiazolidinediones, TriNetX, type 2 diabetes mellitus

## Abstract

**Background:**

Thiazolidinediones are peroxisome proliferator-activated receptor-γ (PPAR-γ) agonists commonly used to treat type 2 diabetes mellitus. In experimental models, epithelial PPAR-γ signaling contributes to colonization resistance against bacterial enteric pathogens by shaping oxygen and nitrate luminal bioavailability. Whether the use of PPAR-γ agonists in humans is associated with reduced risk of bacterial enteric infection is unknown.

**Methods:**

To investigate whether users of thiazolidinediones (PPAR-γ agonists) have decreased risk of bacterial enteric infection, we performed a retrospective nationwide analysis using the cloud-based TriNetX Analytics Platform which aggregates health records from 117 million patients across 66 US healthcare organizations. We compared the risk of bacterial enteric infection among patients with diabetes prescribed thiazolidinediones, a class of PPAR-γ agonists, compared with users of other glucose-lowering medications.

**Results:**

Among 85 916 thiazolidinedione users categorized as being at low risk for bacterial enteric infection (no prior history of infection) and 1974 persons identified as being at high risk (prior history of bacterial enteric infection), the risk of bacterial enteric infection was reduced by 49% (RR 0.51, 95% confidence interval [CI] .32–.82) and 22% (RR 0.78, 95% CI .69–.88) respectively, compared to users of other glucose-lowering medications. This reduction in risk was consistent among high-risk individuals, regardless of sex or age. Similar results were observed in high-risk patients when thiazolidinedione users were directly compared to those on DPP-4 inhibitors.

**Conclusions:**

These findings demonstrate an association between PPAR-γ agonist use and a reduced risk of bacterial enteric infection and support further clinical investigation.

The body's primary defense against bacterial enteric infection relies heavily on the abundant and diverse intestinal microbiota [1, 2]. These gut commensal microbes confer protection through direct bacteria-to-bacteria interactions and activating host immune defenses—a phenomenon known as colonization resistance [1, 2]. As mechanisms of colonization resistance have been elucidated in experimental systems, an important next question is whether these pathways have observable relevance in human populations. Identifying microbial metabolites, proteins, or host receptors involved in preventing infection can lead to new anti-infective strategies and potentially reduce the 1.6–2.1 million annual deaths from diarrheal disease worldwide [3].

Recent mechanistic studies have highlighted a central role of the host nuclear hormone receptor peroxisome proliferator-activated receptor (PPAR-γ) in colonization resistance [4, 5]. Commensal colonic microbes, predominantly obligate anaerobes, thrive in an oxygen- and nitrate-deprived environment, limiting the expansion of facultative anaerobic organisms such as *Salmonella* and other Enterobacteriaceae. These microbes maintain the low-oxygen environment by producing metabolites such as butyrate, which activate PPAR-γ in colonocytes [4, 5]. This activation enhances oxygen consumption by the host, sustaining the protective anaerobic state. In mouse models, depletion of PPAR-γ signaling, triggered by antibiotics or a high-fat diet, has been associated with increased bacterial pathogenesis, aligning with well-known patient risk factors [4–9]. Pharmacological PPAR-γ agonists have also shown promise in restoring this protective effect [10]. While these studies establish a mechanistic framework in animal models, whether pharmacologic modulation of PPAR-γ is associated with altered enteric infection risk in humans has not been examined.

Thiazolidinediones, a class of PPAR-γ agonists, are commonly used to manage type 2 diabetes mellitus (T2DM) [11, 12]. Assessing the risk of bacterial enteric infection among thiazolidinediones users compared users of other glucose-lowering medications could provide insights into the viability of targeting PPAR-γ for anti-infective purposes. In this study, we conducted a nationwide retrospective analysis to assess the risk of bacterial enteric infection among patients with T2DM using thiazolidinediones compared with other glucose-lowering medications.

## METHODS

### Data

We used the cloud-based TriNetX Analytics Platform, US Collaborative Network, to obtain web-based, real-time, secure access to fully deidentified electronic health records of 117 million patients from 66 health care organizations, representing 27% of the US population from all 50 states [13, 14]. Both inpatient and outpatient settings mostly from large academic medical institutions in the US are represented, as well as persons from diverse geographic, age, race and ethnicity, income, and insurance groups. The geographic distribution of patients in the TriNetX platform is 25% in the Northeast, 17% in the Midwest, 41% in the South, and 12% in the West, with 5% unknown.

The TriNetX platform captures data generated during routine clinical care within participating healthcare organizations and was used in this study as an EHR-based dataset only, without supplementation from insurance claims. Available data elements include diagnoses, procedures, medication orders, laboratory results, and healthcare encounters as documented in the contributing institutions' EHRs. Medication exposure reflects provider-entered prescription orders, rather than pharmacy dispensing or adherence. Events occurring outside participating healthcare organizations may not be captured.

### Ethics Statement

Analyses were performed within the TriNetX platform using deidentified individual-level patient records, which enables patient-level analyses such as propensity score matching. Investigators did not receive or export row-level patient data; instead, the platform returns aggregate, population-level outputs. To reduce reidentification risk, TriNetX applies output-level safeguards, including statistical blurring whereby counts of 1 to 10 are reported as 10. Because this study used only deidentified patient records, it was exempt from review by the MetroHealth System Institutional Review Board.

### Study Populations and Risk Stratification

The study period was defined *a priori* as 3 January 2017 through 3 January 2022. The start date was selected to align with contemporary diabetes prescribing patterns, including the introduction of newer agents such as semaglutide for the management of T2DM (FDA-approved in December 2017). The end date was selected to minimize overlap with expanded GLP-1 agonist indications for weight loss in individuals without diabetes (FDA-approved in June 2021), which would introduce heterogeneity in prescribing indications.

Adults ≥ 18 years of age with a diagnosis of type 2 diabetes mellitus (T2DM) who, within one month of a diabetes-related clinical encounter during the study period, had a provider-entered prescription order for either (i) a thiazolidinedione (pioglitazone or rosiglitazone) or (ii) a nonthiazolidinedione glucose-lowering medication (GLP-1 receptor agonist, insulin, metformin, sulfonylureas, α-glucosidase inhibitors, DPP-4 inhibitors, or SGLT2 inhibitors) were eligible for inclusion. Troglitazone users were excluded because the drug is no longer available in the United States. Individuals with any thiazolidinedione prescription during the study period were excluded from the nonthiazolidinedione comparison cohort. The index event was defined as the date of the qualifying glucose-lowering medication prescription order.

Risk for bacterial enteric infection can often be influenced by factors that are not well captured by electronic medical records (eg, travel, food exposures, outbreaks, antibiotic use, and other environmental and behavioral factors). To account for heterogeneity in baseline susceptibility, the study population was stratified a priori based on a documented history of bacterial enteric infection before the index event. Low-risk individuals were defined as those without a diagnosis of bacterial enteric infection before the index prescription. High-risk individuals were defined as those with a documented bacterial enteric infection before the index prescription. Prior infection was used as an empirical marker of elevated baseline risk rather than as a mechanistic determinant, reflecting a constellation of host, environmental, and healthcare-associated factors that may predispose to recurrent exposure, infection, or diagnosis and cannot be fully measured in EHR-based datasets. The stratification approach is consistent with prior molecular-epidemiologic assessments and outbreak investigations describing risk patterns for bacterial enteric infections [15–18]. All subsequent analyses were performed separately within each of these risk strata.

### Statistical Analysis

For each study cohort, the thiazolidinedione users and nonthiazolidinedione users were propensity score matched (1:1 using nearest-neighbor greedy matching with a caliper of 0.25 times the standard deviation) on covariates that are potential risk factors for bacterial enteric infection including diabetes severity, age, sex, race, ethnicity, overweight and obesity, lifestyle problems, ischemic heart disease, liver disease, kidney disease, lung disease, hypertension, hyperlipidemia, cerebrovascular disease, atherosclerosis, other peripheral vascular disease, tobacco use, alcohol use, human immunodeficiency virus (HIV), organ transplant, use of immunosuppressants, use of chemotherapeutics, and hemoglobin (Hgb) A1c levels.

The occurence of bacterial enteric infection during the 6-month window after the index event (prescription of medication) was compared between the matched medication groups. The status of bacterial enteric infection was defined by ICD-10 codes A00-A05 (A00: Cholera, A01: Typhoid and paratyphoid fevers, A02: Other salmonella infections, A03: Shigellosis, A04: Other bacterial intestinal infections, A05: Other bacterial foodborne intoxications, not elsewhere classified). Because counts of bacterial enteric infection were low in the cohort, analyses of risk associated with individual bacterial species (ie, *Salmonella* sp, enterohemorrhagic *E. coli*) precluded statistical analysis (most counts were fewer than 10, therefore were reported as 10 by TriNetX) and were not performed. Only a composite outcome was investigated.

Several subgroup analyses were performed to assess the robustness of our results and possible bias. First, outcomes were examined when stratified by sex and age. Second, because diabetes medications are often prescribed in combination, it was necessary to evaluate whether other medications commonly administered alongside thiazolidinediones could confound observed effects. However, thiazolidinediones are rarely used for the management of T2DM because of weight gain, pedal edema, and heart failure exacerbation, leading to their classification as a third-line option. Accordingly, thiazolidinediones were compared with another third-line class of glucose-lowering medications, dipeptidyl peptidase-4 (DPP-4) inhibitors [12]. Age- and sex-stratified analyses were not performed between thiazolidinediones and DPP-4 inhibitors as subgroups often had fewer than 10 persons with the desired outcome. To maintain deidentification, TriNetX does not provide actual counts below 10, which precludes accurate statistical analysis. The primary and sensitivity analyses were conducted in TriNetX between August 4 and August 11, 2024. Because the TriNetX network is continuously updated, each analysis used the network data available when the corresponding query was executed. The same cohort definitions, inclusion criteria, and analytic parameters were used for each analysis. All statistical analyses were performed with the TriNetX Advanced Analytics Platform using built-in functions (R software, version 3.2-3; R program for Statistical Computing), with statistical significance defined as a 95% confidence interval (CI) excluding 1. Standard mean differences > 0.1 were considered to indicate a group imbalance.

## RESULTS

### Association of Thiazolidinedione Use With Risk of Bacterial Enteric Infection in Patients at Low Risk

The study population consisted of 85 917 and 2 154 436 persons with T2DM who between January 2017 and January 2022 were prescribed a thiazolidinedione or a nonthiazolidinedione glucose-lowering medications, respectively, and had no prior history of bacterial enteric infection. Persons on thiazolidinediones compared with those on nonthiazolidinedione gluose-lowering medications were older, more likely to be male and White, and had a higher prevalence of overweight and obesity, chronic kidney disease, and Hgb A1c levels of 9% or greater (Table 1).

**Table 1.** Characteristics of the Thiazolidinedione and the Nonthiazolidinedione Glucose-Lowering Medication Cohorts for the Study Population at Low Risk for Bacterial Enteric Infection

	Before matching			After Matching		
Characteristics	TZD users	Non-TZD users	Std diff.	TZD users	Non-TZD users	Std diff.
Total no.	85 917	2 154 436		85 916	85 916	…
Age, mean, y	63.2 ± 12.3	61.4 ± 14.5	0.13	63.2 ± 12.3	63.4 ± 12.8	0.014
Sex, %						
Male	54.9 (47 127)	49.0 (1 055 494)	0.117	54.9 (47 126)	55.5 (47 699)	0.013
Female	42.9 (36 833)	46.9 (1 011 390)	0.082	42.9 (36 833)	42.3 (36 342)	0.012
Ethnicity, %						
Hispanic/Latino	14.3 (12 297)	9.3 (200 454)	0.156	14.3 (12 296)	13.6 (11 666)	0.021
Not Hispanic/Latino	64.2 (55 175)	62.3 (1 342 333)	0.040	64.2 (55 175)	65.1 (55 969)	0.019
Unknown	21.5 (18 445)	28.4 (611 649)	0.161	21.5 (18 445)	21.3 (18 281)	0.005
Race, %						
African American/Black	12.8 (10 982)	18.0 (387 328)	0.144	12.8 (10 982)	12.8 (10 958)	0.001
Asian	5.1 (4411)	4.4 (94 934)	0.034	5.1 (4411)	5.1 (4383)	0.001
White	67.7 (58 193)	59.9 (1 289 841)	0.164	67.7 (58, 192)	68.1 (58 527)	0.008
American Indian or Alaska Native	0.3 (253)	0.3 (6135)	0.002	0.3 (253)	0.3 (264)	0.002
Native Hawaiian or other Pacific Islander	0.4 (310)	0.6 (12 633)	0.033	0.4 (310	0.3 (273)	0.007
Other race	3.4 (2935)	3.4 (72 733)	0.002	3.4 (2935)	3.3 (2800)	0.009
Unknown	10.3 (8833)	13.5 (290 832)	0.100	10.3 (8833)	10.1 (8711)	0.005
Problems related to lifestyle	4.5 (3894)	4.0 (87 574)	0.023	4.5 (3894)	4.1 (3482)	0.024
Tobacco use	10.0 (8623)	10.7 (230 612)	0.022	10.0 (8623)	9.1 (7857)	0.030
Alcohol use disorder	2.1 (1765)	2.8 (60 514)	0.049	2.1 (1765)	1.8 (1562)	0.017
Comorbidities, %						
Overweight and obesity	31.0 (26 652)	25.0 (538 806)	0.134	31.0 (26 651)	30.1 (25 881)	0.019
BMI <20	0.3 (298)	0.5 (11 256)	0.027	0.3 (298)	0.3 (260)	0.008
BMI 20–29	4.8 (4145)	4.0 (85 378)	0.042	4.8 (4145)	4.4 (3809)	0.019
BMI 30–39	12.3 (10 591)	9.2 (10 591)	0.100	12.3 (10 590)	11.9 (10 225)	0.013
BMI ≥40	7.2 (6225)	6.3 (6225)	0.037	7.2 (6225)	7.0 (5988)	0.011
Hypertension	64.0 (55 024)	51.6 (1 111 828)	0.254	64.0 (55 023)	63.5 (54 584)	0.011
Hyperlipidemia	48.4 (41 614)	35.5 (765 392)	0.264	48.4 (41 613)	47.7 (40 956)	0.015
Chronic respiratory disease	17.0 (14 591)	17.1 (368 913)	0.004	17.0 (14 591)	16.3 (14 023)	0.018
Liver disease	10.2 (8775)	8.6 (184 475)	0.057	10.2 (8775)	9.5 (8143)	0.025
Chronic kidney disease	14.0 (12 020)	12.6 (272 199)	0.040	14.0 (12 020)	13.2 (11 351)	0.023
CKD stage 1	0.8 (662)	0.5 (11 300)	0.031	0.8 (662)	0.7 (573)	0.012
CKD stage 2 (mild)	2.5 (2144)	1.8 (38 754)	0.048	2.5 (2144)	2.2 (1915)	0.018
CKD stage 3 (moderate)	12.3 (7860)	9.2 (148 619)	0.083	9.1 (7860)	8.7 (7480)	0.016
CKD stage 4 (severe)	1.9 (1665)	2.2 (46 647)	0.016	1.9 (1665)	1.8 (1539)	0.011
CKD stage 5	0.5 (449)	1.0 (21 425)	0.054	0.5 (449)	0.5 (428)	0.003
End stage renal disease	1.2 (1069)	2.7 (58 452)	0.106	1.2 (1069)	1.2 (1015)	0.006
Ischemic heart disease	17.0 (14 624)	18.4 (395 753)	0.035	17.0 (14 624)	16.3 (14 007)	0.019
Cerebrovascular disease	8.7 (7507)	9.1 (195 269)	0.011	8.7 (7507)	8.1 (6959)	0.023
Atherosclerosis	4.5 (3907)	4.1 (98 303)	0.001	4.5 (3907)	4.1 (3562)	0.020
Other peripheral vascular disease	5.7 (4930)	5.2 (112 598)	0.022	5.7 (4930)	5.1 (4421)	0.026
HIV	0.6 (493)	0.5 (10 556)	0.012	0.6 (493)	0.5 (436)	0.009
Organ transplant	0.9 (794)	1.6 (34 361)	0.060	0.9 (344)	0.9 (381)	0.006
Medications, %						
Immunosuppressants	3.2 (2745)	3.5 (75 339)	0.017	3.2 (2745)	3.0 (2568)	0.012
Chemotherapy	12.4 (10 670)	11.4 (246 333)	0.030	12.4 (10 670)	12.0 (10 287)	0.014
Labs, %						
Hgb A1c						
<9%	54.3 (46 688)	43.2 (930 268)	0.225	54.3 (46 687)	54.0 (46 416)	0.006
≥9%	30.6 (26 317)	16.5 (355 020)	0.338	30.6 (25 892)	30.1 (25 892)	0.011

Abbreviations: TZD, thiazolidinedione; non-TZD, nonthiazolidinedione; std diff, standardized mean difference; y, years; CKD, chronic kidney disease; HIV, human immunodeficiency virus; BMI, body mass index.

**Figure 1.**
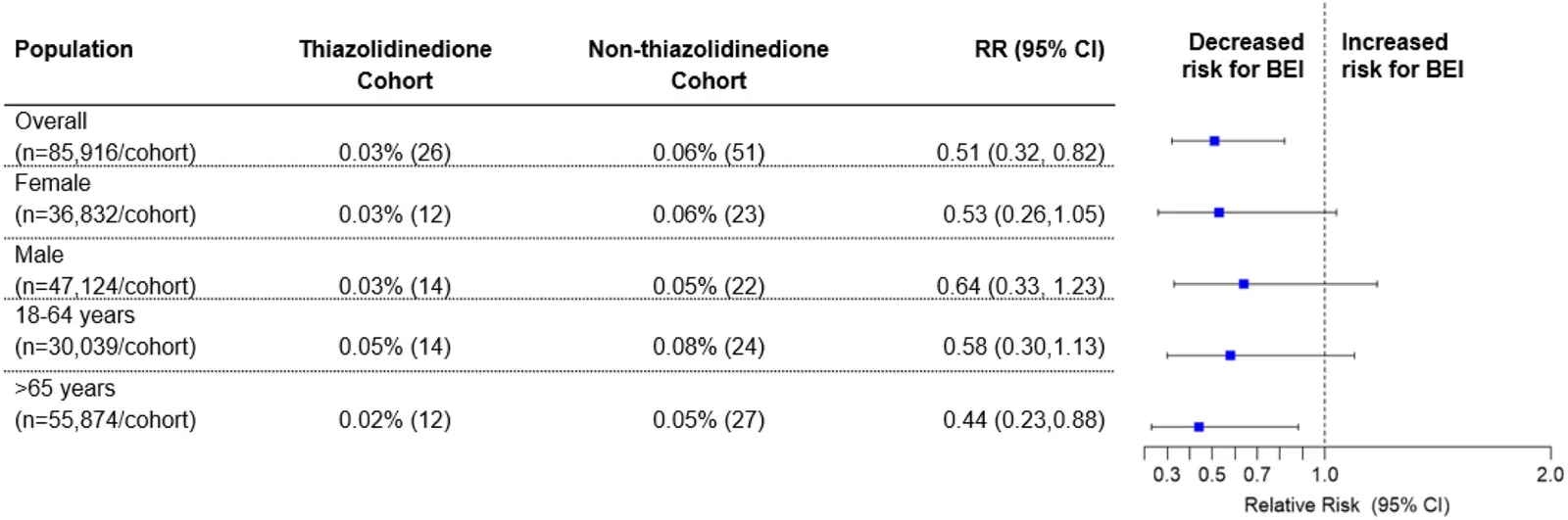
Risk of bacterial enteric infection in low-risk individuals; BEI, bacterial enteric infection.

After 1:1 propensity matching, demographic and clinical characteristics were balanced (85 916 in each cohort, mean age, 63.2 years; 42.9% female, 12.8% Black, 67.7% White, 14.3% Hispanic) (Table 1). Matched cohorts were followed for 6 months after the index event. Compared to nonthiazolidinedione glucose-lowering medication use, thiazolidinedione use was associated with a lower risk of incident bacterial enteric infection among those at low risk (0.03% vs 0.06%; RR, 0.51; 95% CI, 0.32-0.82). An association with a lower risk for bacterial enteric infection was observed across age and sex subgroups; however, findings were statistically significant only among those >65 years (0.02% vs 0.05%; RR, 0.44; 95% CI 0.23-0.88) (Figure 1).

### Association of Thiazolidinedione Use With Risk of Bacterial Enteric Infection in Patients at High Risk

The study population consisted of 1975 and 57 963 persons with T2DM who between January 2017 and January 2022 were prescribed thiazolidinedione or nonthiazolidinedione glucose-lowering medications, respectively, and had a prior history of bacterial enteric infection. Persons on thiazolidinediones were more likely to be Hispanic or White, and had a higher prevalence of overweight and obesity, hypertension, hyperlipidemia, and Hgb A1C levels ≥9.

**Figure 2.**
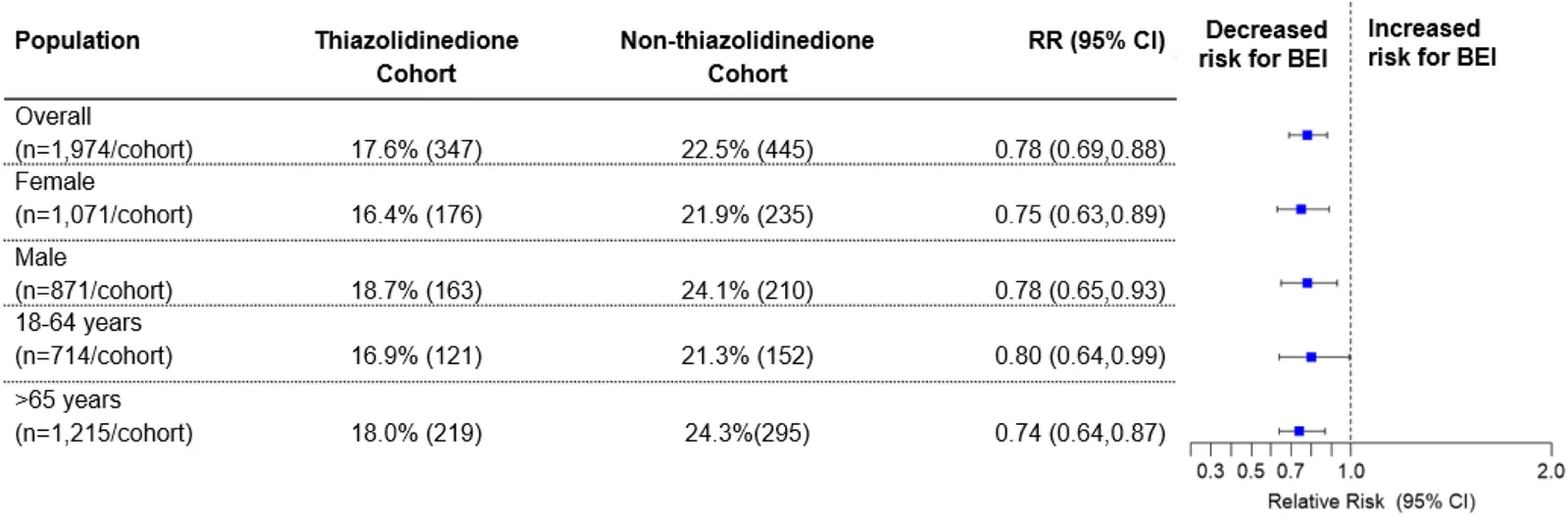
Risk of bacterial enteric infection in high-risk individuals; BEI, bacterial enteric infection.

After 1:1 propensity matching, demographic and clinical characteristics were balanced (1974 in each cohort, mean age, 63.0; 44.1% female, 16.9% Black, 65.4% White, 20.8% Hispanic) (Table 1). Matched cohorts were followed for 6 months after the index event. Compared to nonthiazolidinedione glucose-lowering medication use, thiazolidinedione use was associated with a lower risk of incident bacterial enteric infection among those at high risk (17.6% vs 22.5%; RR, 0.78; 95% CI 0.690.88). An association with a lower risk for bacterial enteric infection was observed across all sex and age subgroups (Figure 2).

### Association of Thiazolidinedione Use Compared With DPP-4 Inhibitors Use With Risk of Bacterial Enteric Infection in Patients at Low and High Risk (Sensitivity Analysis)

**Figure 3.**
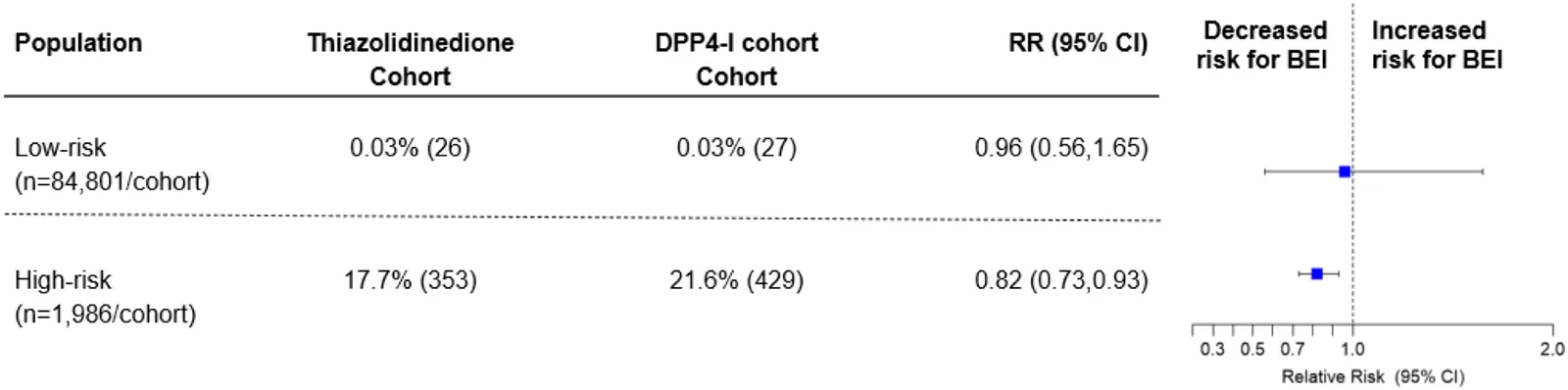
Risk of bacterial enteric infection in patients with T2DM using thiazolidinedione compared with those using DPP-4 inhibitors at low risk and high risk; BEI, bacterial enteric infection, T2DM, type 2 diabetes mellitus.

In the matched cohorts (Table 2), thiazolidinedione use, compared with DPP-4 inhibitor use, was associated with a lower risk of bacterial enteric infection in those at high risk (RR 0.82; 95% CI 0.72, 0.93) and not among those at low risk (RR, 0.96; 95% CI 0.56-1.65) (Figure 3).

**Table 2.** Characteristics of the Thiazolidinedione and Nonthiazolidinedione Glucose-Lowering Medication Cohorts for the Study Population at High Risk for Bacterial Enteric Infection

	Before matching			After Matching		
Characteristics	TZD users	Non-TZD users	Std diff.	TZD users	Non-TZD users	Std diff.
Total No.	1975	57 963	…	1974	1974	…
Age, mean, y	63.0 ± 12.7	62.6 ± 14.1	0.029	63.0 ± 12.7	63.1 ± 13.3	0.007
Sex, %						
Male	44.1 (871)	43.3 (25 086)	0.017	44.1 (871)	42.8 (845)	0.027
Female	54.3 (1072)	52.9 (30 635)	0.029	54.3 (1071)	55.1 (1088)	0.017
Ethnicity, %						
Hispanic/Latino	20.9 (412)	12.3 (7103)	0.233	20.8 (411)	20.8 (410)	0.001
Not Hispanic/Latino	65.7 (1297)	66.3 (38 434)	0.013	65.7 (1297)	64.5 (1273)	0.026
Unknown	13.5 (266)	21.4 (12 426)	0.211	13.5 (266)	14.7 (291)	0.036
Race, %						
African American/Black	16.9 (334)	20.8 (12 041)	0.099	16.9 (334)	16.3 (322)	0.016
Asian	4.4 (87)	4.1 (2394)	0.014	4.4 (87)	4.4 (87)	<0.001
White	65.4 (1292)	57.7 (33 471)	0.158	65.4 (1291)	65.8 (1299)	0.009
American Indian or Alaska Native	0.5 (10)	0.4 (217)	0.020	0.5 (10)	0.5 (10)	<0.001
Native Hawaiian or other Pacific Islander	0.6 (11)	1.0 (597)	0.053	0.6 (11)	0.5 (10)	0.007
Other Race	3.4 (68)	3.3 (1885)	0.011	3.4 (68)	3.2 (64)	0.011
Unknown	9.0 (177)	12.7 (7358)	0.120	9.0 (177)	9.4 (185)	0.014
Lifestyle problem	8.6 (169)	7.6 (4398)	0.036	8.6 (169)	7.4 (147)	0.041
Tobacco use	16.2 (320)	18.2 (10 551)	0.053	16.2 (320)	14.4 (285)	0.049
Alcohol use disorder	5.3 (104)	6.9 (3976)	0.067	5.3 (104)	4.4 (87)	0.040
Comorbidities, %						
Overweight and obesity	45.9 (906)	38.6 (22 388)	0.147	45.8 (905)	43.4 (857)	0.049
BMI <20	1.8 (36)	2.0 (1143)	0.011	1.8 (36)	1.6 (31)	0.020
BMI 20–29	8.4 (165)	8.4 (4851)	0.001	8.4 (165)	7.0 (139)	0.049
BMI 30–39	17.5 (345)	15.2 (8841)	0.061	17.5 (345)	16.0 (316)	0.039
BMI ≥40	12.7 (250)	10.5 (6099)	0.067	12.6 (249)	11.6 (229)	0.031
Hypertension	79.8 (1577)	71.5 (41 469)	0.195	79.8 (1576)	77.1 (1522)	0.067
Hyperlipidemia	67.4 (1331)	54.7 (31 679)	0.263	67.4 (1330)	64.9 (1282)	0.051
Chronic respiratory disease	29.6 (584)	31.9 (18 464)	0.050	29.6 (584)	27.7 (546)	0.043
Liver disease	24.4 (481)	21.8 (12 622)	0.061	24.2 (481)	22.2 (438)	0.052
Chronic kidney disease	28.7 (566)	32.5 (18 864)	0.084	28.6 (565)	27.4 (541)	0.027
CKD stage 1	2.0 (39)	1.5 (873)	0.036	1.9 (38)	2.3 (46)	0.028
CKD stage 2 (mild)	5.7 (113)	5.1 (2980)	0.026	5.7 (113)	6.3 (125)	0.026
CKD stage 3 (moderate)	19.3 (381)	18.2 (10 533)	0.029	19.3 (380)	18.2 (359)	0.027
CKD stage 4 (severe)	5.4 (107)	8.6 (4992)	0.125	5.4 (107)	5.1 (101)	0.014
CKD stage 5	1.7 (34)	5.0 (2917)	0.184	1.7 (34)	1.6 (31)	0.012
End stage renal disease	4.4 (86)	10.9 (6314)	0.248	4.4 (86)	4.0 (78)	0.020
Ischemic heart disease	28.7 (566)	33.9 (19 634)	0.113	28.7 (566)	26.2 (518)	0.055
Cerebrovascular disease	17.0 (335)	18.9 (10 935)	0.050	16.9 (334)	14.6 (288)	0.064
Atherosclerosis	10.9 (215)	12.2 (7100)	0.043	10.9 (215)	9.2 (182)	0.056
Other peripheral vascular disease	11.6 (229)	12.8 (7413)	0.037	11.6 (229)	10.0 (198)	0.051
HIV	1.4 (28)	1.3 (774)	0.007	1.4 (28)	1.3 (26)	0.009
Organ transplant	3.7 (44)	8.0 (3128)	0.184	3.7 (46)	4.0 (78)	0.013
Medications						
Immunosuppressants	8.2 (162)	11.7 (6757)	0.116	8.2 (162)	7.0 (139)	0.044
Chemotherapy	24.8 (489)	25.6 (14 863)	0.020	24.8 (489)	22.7 (448)	0.049
Labs						
Hgb A1c						
<9%	69.8 (1378)	61.6 (35 705)	0.173	69.8 (1377)	69.1 (1377)	0.014
≥9%	43.0 (849)	24.5 (14 191)	0.400	43.0 (849)	43.2 (852)	0.003

Abbreviations: TZD, thiazolidinedione; non-TZD, nonthiazolidinedione; std diff, standardized mean difference; y, years; CKD, chronic kidney disease; HIV, human immunodeficiency virus; BMI, body mass index.

## DISCUSSION

Our analysis identified a significant association between thiazolidinedione use and a reduced risk of bacterial enteric infection in T2DM patients, consistent with findings from animal models. This association was observed in both cohorts across age and sex subgroups among individuals with a history of bacterial enteric infection. The association with reduced risk was also seen when comparing thiazolidinediones with another class of third-line glucose-lowering medications, DPP-4 inhibitors. Together, these findings suggest that a clinically used class of PPAR-γ agonists is associated with reduced bacterial enteric infection in a large real-world population and support further investigations of PPAR-γ-related pathways in human enteric infection susceptibility.

Thiazolidinediones are typically used as third-line medications when other glucose-lowering medications have proven ineffective or when insurance limitations restrict alternative options [12]. Consequently, thiazolidinedione users often represent a population with more severe or poorly controlled diabetes, characterized by frequent healthcare encounters and lower socioeconomic status [19, 20]. These factors are associated with an increased risk of bacterial enteric infection, suggesting that the protective effect of thiazolidinediones may be greater than that indicated by this analysis [21–23].

Repurposing thiazolidinediones is not a novel concept. Initially, PPAR-γ was believed to primarily function in adipose tissue, regulating insulin resistance, which led to the development of thiazolidinediones for diabetes management [24]. However, investigators soon appreciated the high expression of PPAR-γ in colonic epithelial cells and its involvement in colon cancer [25, 26]. Subsequently, PPAR-γ signaling was shown to reduce colonic inflammation, prompting the exploration of thiazolidinediones for inflammatory bowel disease [27]. Promising results were observed in mouse models and in clinical trials [28, 29]. Despite these benefits, concerns over toxicities, such as weight gain, fluid retention, and heart failure, have overshadowed the benefits of thiazolidinediones. Therefore, while this study suggests thiazolidinediones may protect against bacterial enteric infection, their repurposing for this indication will likely be limited by their associated risks.

Fortunately, there are alternative ways to increase PPAR-γ signaling. For instance, mesalamine (5-ASA), a PPAR-γ agonist widely used for treating mild to moderate ulcerative colitis, may offer a safer alternative. Studies suggest that 5-ASA prevents the expansion of colitogenic bacteria such as *Escherichia coli* [10], suggesting its potential role in preventing bacterial enteric infection in high-risk individuals.

Dietary modifications may also enhance PPAR-γ signaling. A high-fiber diet can increase butyrate levels, which, in turn, activates PPAR-γ. However, this depends on the presence of a gut microbiota capable of producing butyrate. Unsaturated fatty acids, including those found in dairy and meat products, are natural PPAR-γ ligands and may also promote colonic health, although achieving sufficient intraluminal concentrations *in vivo* remains controversial. Small animal studies have demonstrated possible benefits from such dietary interventions [30, 31].

One hypothesis for the protective effects of PPAR-γ activation in mouse models is its ability to maintain an anaerobic environment in the gut, which helps prevent bacterial enteric infections. However, this mechanism does not directly explain why thiazolidinediones may also protect against infection from *Clostridioides difficile*, an obligate anaerobe. It is possible that PPAR-γ signaling supports the growth of beneficial anaerobes such as *Clostridium scindens* or *Blautia producta*, which inhibit *C. difficile* through production of secondary bile acids and lantibiotics, respectively [32, 33]. Furthermore, PPAR-γ activity in macrophages, which has been shown to ameliorate colonic inflammation, may also contribute to this protective effect. Given that thiazolidinediones are primarily absorbed in the small intestine and may not reach the colon directly, this macrophage-mediated mechanism merits further investigation [10].

### Limitations

There are limitations to consider when interpreting the results of this study. First, this retrospective analysis identified associations and cannot establish causality. Residual confounding, particularly that related to diabetes severity, may have influenced the findings. Although we controlled for factors such as ischemic heart disease, peripheral vascular disease, chronic kidney disease, and Hgb A1c levels, fully characterizing the severity of diabetes remains a challenge. Further, medication adherence cannot be confirmed based on electronic health records. However, our inclusion of individuals with thiazolidinedione or other glucose-lowering prescriptions following a diabetes-related healthcare encounter suggests active management of the disease. Low event counts precluded an analysis of dose-dependent effects and we could not assess the duration of thiazolidinedione use required for a protective effect. Furthermore, because T2DM is often managed with a combination of medications, it is possible that other medications commonly used alongside thiazolidinediones contributed to the observed protective effect. To address this, we directly compared thiazolidinedione users with DPP-4 inhibitor users; this analysis showed a lower risk of bacterial enteric infection among high-risk thiazolidinedione users. Finally, the severity of bacterial enteric infections was not captured in this study, so it remains unknown whether thiazolidinediones can reduce disease severity.

## CONCLUSION

Bacterial enteric infections remain a significant public health concern, with acute gastroenteritis causing an estimated 179 million outpatient visits and nearly 500 000 hospitalizations annually [34–36]. The use of current antimicrobial treatments are often limited, because antibiotics can prolong symptoms, increase bacterial shedding, and contribute to recurrence in some settings [15–18]. Given these limitations, strategies that preserve beneficial intestinal microbes and minimize collateral effects on the microbiome remain an important area of investigation. In this context, the observed association between thiazolidinedione use and a lower risk of bacterial enteric infection supports further prospective and mechanistic studies of PPAR-γ-related pathways in human enteric infection susceptibility.
